# CPS-Net (Collaborating Physicians in Silico Network): A Decentralized Multi-Agent Transformer Framework for Specialty-Aware Disease Prediction

**DOI:** 10.1007/s10916-026-02460-8

**Published:** 2026-09-15

**Authors:** Mohammad Assadi Shalmani, Masoud Khani, Michael S. Harris, Qiang Lu, Jake Luo

**Affiliations:** 1 Health Informatics Program, Zilber School of Public Health, University of Wisconsin-Milwaukee, Milwaukee, WI USA; 2 Department of Otolaryngology & Communication Sciences, Medical College of Wisconsin, Milwaukee, WI USA; 3 Department of Computer Science and Technology, China University of Petroleum, Beijing, China

**Keywords:** Large Language Models, Agent Swarm, Transformer, Disease Diagnosis, Electronic Health Records

## Abstract

Diagnosis in real clinical settings is rarely finalized after a single encounter. Patients with complex conditions move through a referral network from primary care to specialists because physicians cannot maintain expertise across all diseases. Yet most diagnostic prediction tools apply a single generalist model across thousands of medical codes, which dilutes representational capacity for rare conditions and produces outputs poorly aligned with how clinicians actually work and reason. We present CPS-Net, a decentralized agent swarm that mirrors medical specialization hierarchies. The system connects specialized transformers trained on focused patient cohorts to language model agents organized into primary care, specialty, and subspecialty tiers. Agents collaborate without central supervision, communicating through shared case memory to make referral decisions transparent and auditable. We trained 41 transformers on Electronic Health Records (EHR) from 75,000 patients across 10 specialties and 30 subspecialties at Froedtert Hospital. Evaluated on 3,000 test cases, the framework achieved top-1, top-3, and top-5 accuracies of 48.8%, 71.00%, and 77.76% with 94.17% clinical relevance, substantially outperforming a monolithic transformer with 19.20%, 38.60% and 47.20% in top-1, top-3 and top-5 accuracy trained on all patients and a single language model agent baseline which achieved 12.70%, 26.40%, and 37.23% accuracy. This work demonstrates that aligning artificial intelligence architecture with clinical workflows improves both prediction accuracy and interpretability for diagnostic support.

**Supplementary Information:**

The online version contains supplementary material available at https://doi.org/10.1007/s10916-026-02460-8.

## Introduction

A patient’s treatment journey typically begins with a primary care physician who generally examines patients, reviews the medical history and orders necessary tests [1,2]. Many diseases are managed at this stage, but when a condition falls outside primary care expertise, they refer the patient to a specialist [3,4]. Specialists focus on specific disease categories [5], and the referral depends on which specialty is most likely to address the patient’s health issue. For particularly complex conditions, there might be a need for specialists who have developed knowledge in narrow disease domains through dedicated experience on specific conditions [6].

Nowadays diagnosing patients often requires input from multiple physicians before a diagnosis is finalized [7]. This structure arises from the high granularity of medical knowledge, which makes it impossible for any clinician to maintain current expertise across all diseases [8]. Diagnosis in current clinical setting is therefore decomposed from a general assessment in primary care to a specific diagnosis at the specialty level [9].

However, machine learning tools have increasingly been applied to support clinical diagnosis, current approaches to developing predictive models do not simulate actual hospital settings [10]. Predictive models have enabled diagnosis prediction from structured data while early approaches relied solely on manually engineered features [11,12]. These models also struggle when the number of features expands significantly [13]. More importantly, they are unable to process sequences of events over time, which is essential for understanding disease behavior [14].

Hence, deep learning sequence models, particularly recurrent neural networks and their variants such as LSTMs and GRUs, were developed to operate on sequences of visits to predict future diagnoses [15]. However, these models convert prediction into a classification task, determining whether a target disease is likely to occur in a patient’s future [16]. Moreover, they struggle with long patient histories, typically flattening complex patient trajectories due to memory constraints [17]. Studies have also noted that they are limited to traditional external validation approaches [18].

In contrast, transformers are defined as effective tools for sequence modeling through their self-attention mechanism [19]. This capability makes transformers particularly well suited for capturing long-range dependencies in medical data [20]. Applied to electronic health records, transformers treat medical codes as tokens ordered by time [21]. Compared to traditional machine learning and deep learning models, transformers handle long sequences spanning years more effectively and offer more flexible modeling of disease interactions [20, 22]. These properties are valuable because patient histories contain events with complex temporal relationships [23].

Despite these strengths, most existing work uses a single global transformer trained on all patients and all medical codes in one attention space. This approach produces a general model that may not accurately predict across different disease types, suggesting room for systems that better align with hospital workflows. Moreover, many medical codes occur rarely, resulting in extreme data sparsity that can lead to biased predictions [24]. Beyond technical challenges, these models output a diagnostic label rather than a decision path that clinicians can follow and validate. To better support healthcare providers in diagnosis, models need to explain the reasoning behind the diagnosis [25].

In the meantime, large language models have demonstrated the ability to generate clinical summaries and facilitate patient-physician communication [26]. With recent advancements in artificial intelligence, individuals are increasingly using large language models for medical consultation [27]. These models offer general-purpose language-based reasoning and can explain their decisions in natural language. However, they can hallucinate, exhibit overconfidence, and fail when a patient’s condition differs from their general training distribution [28]. Additionally, most LLM applications focus on text inputs and often ignore the structured, longitudinal sequences present in electronic health records, which contain comprehensive information on patient medical history [29, 30]. In response, agentic frameworks have advanced rapidly. By decomposing tasks so that each agent specializes in a specific part, the framework can solve complex problems by breaking them into manageable components [31]. Agentic frameworks have shown improvements over other approaches in prediction, consultation, and interpretation [32, 33].

In summary, two fundamental gaps persist in current approaches to AI-supported clinical diagnosis. First, existing EHR-based models such as transformers and LLMs treat diagnosis prediction as a monolithic task, rather than mirroring the hierarchical structure of medical practice which limits their practical utility. Second, they function as models that provide predictions without clear reasoning. To evaluate and address these gaps, we developed three research questions. First, can connecting specialized transformers, to large language models which are trained on general text and medical information provide accurate diagnosis prediction? Second, does decomposing the diagnostic task into primary care, specialty-level, and subspecialty-level predictions improve next-disease prediction compared with current predictive approaches? Third, does an orchestrator-free multi-agent framework, where agents consult in natural language, increase interpretability and trust relative to single-model or centrally orchestrated baselines?

Therefore, we designed a hierarchical agent swarm which mirrors the structure of clinical practice. We defined a primary care agent that processes the full patient history and connects to a transformer model trained on a general set of clinical codes. This agent identifies which specialty the patient should visit. The primary care is connected to a set of specialists, each connected to a transformer trained on a more specific set of codes. For higher accuracy, we defined highly specific trained agents called subspecialist agents which are connected to transformers trained on specific exact ICD-10 codes. These agents can either provide a diagnosis or redirect to another subspecialist when they feel it is more appropriate. Critically, the system operates without a central supervisor or router. All agents interact through a shared case memory that stores patient history, predictions, and explanations, and every referral and justification appear explicitly in the dialogue. This unsupervised architecture makes the full reasoning chain transparent for clinicians and improves the auditability of the diagnostic process.

## Methods

**Fig. 1:**
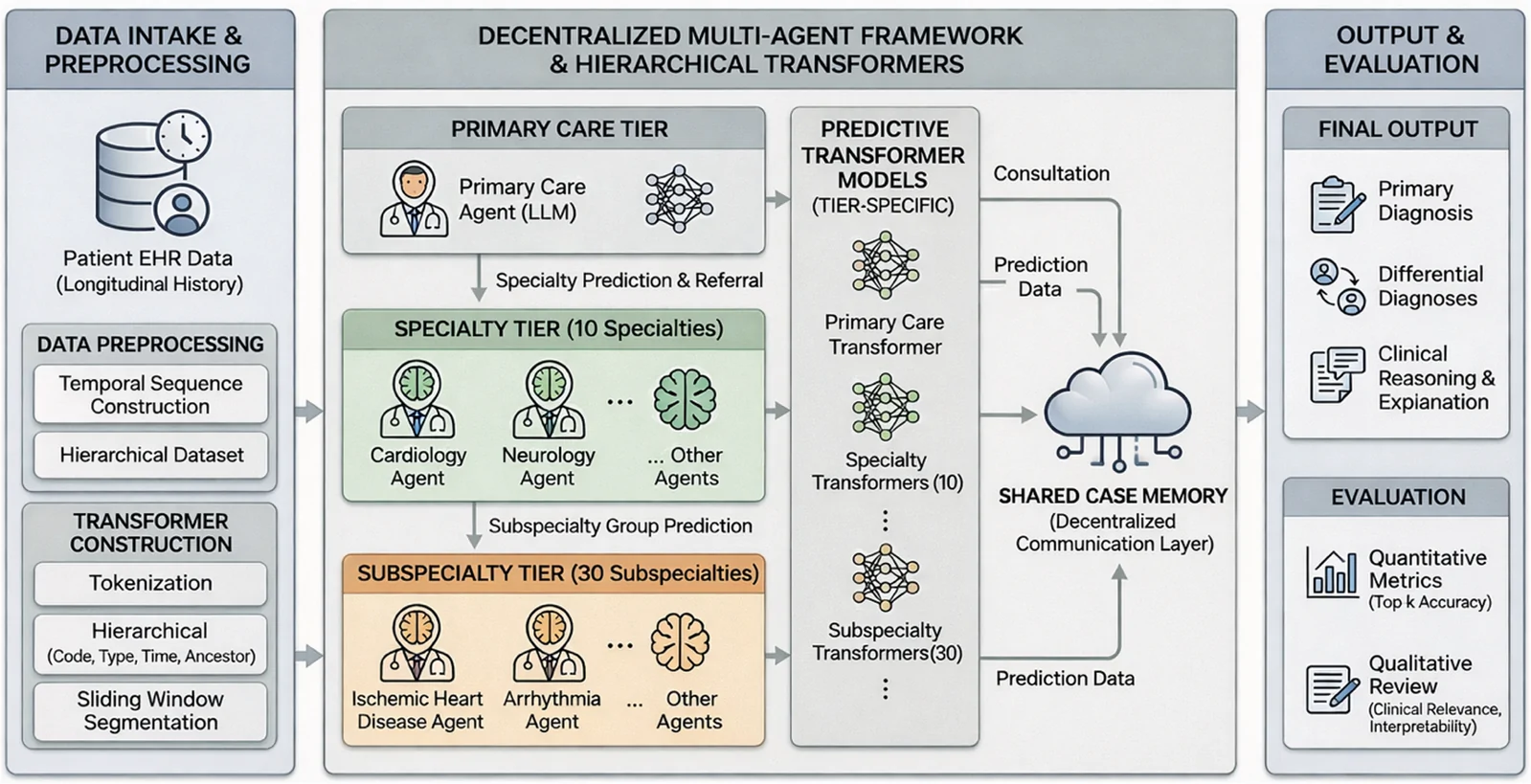
Overview of the CPS-Net for specialty-aware diagnosis prediction

CPS-Net mirrors real-world clinical diagnosis, where patients move through a referral network from primary care to specialists. Figure 1 illustrates the overall architecture, consisting of three main stages: data intake and preprocessing, the multi-agent framework with hierarchical transformers, and output evaluation.

The agentic system is organized into three tiers: a Primary Care Tier that processes patient histories and makes referrals, a Specialty Tier with 10 agents representing major medical fields (e.g., Cardiology, Neurology), and a Subspecialty Tier with 30 highly specialized agents. Through shared case memory, the transformer models with the help of the agent swarm collectively refer patients and select the most appropriate specialist and predictive model for each case. Each tier uses transformers trained on data at the corresponding level of specificity, combining broad medical knowledge with deep subspecialty expertise.

### Data Collection and Preprocessing

#### EHR Cohort

**Fig. 2:**
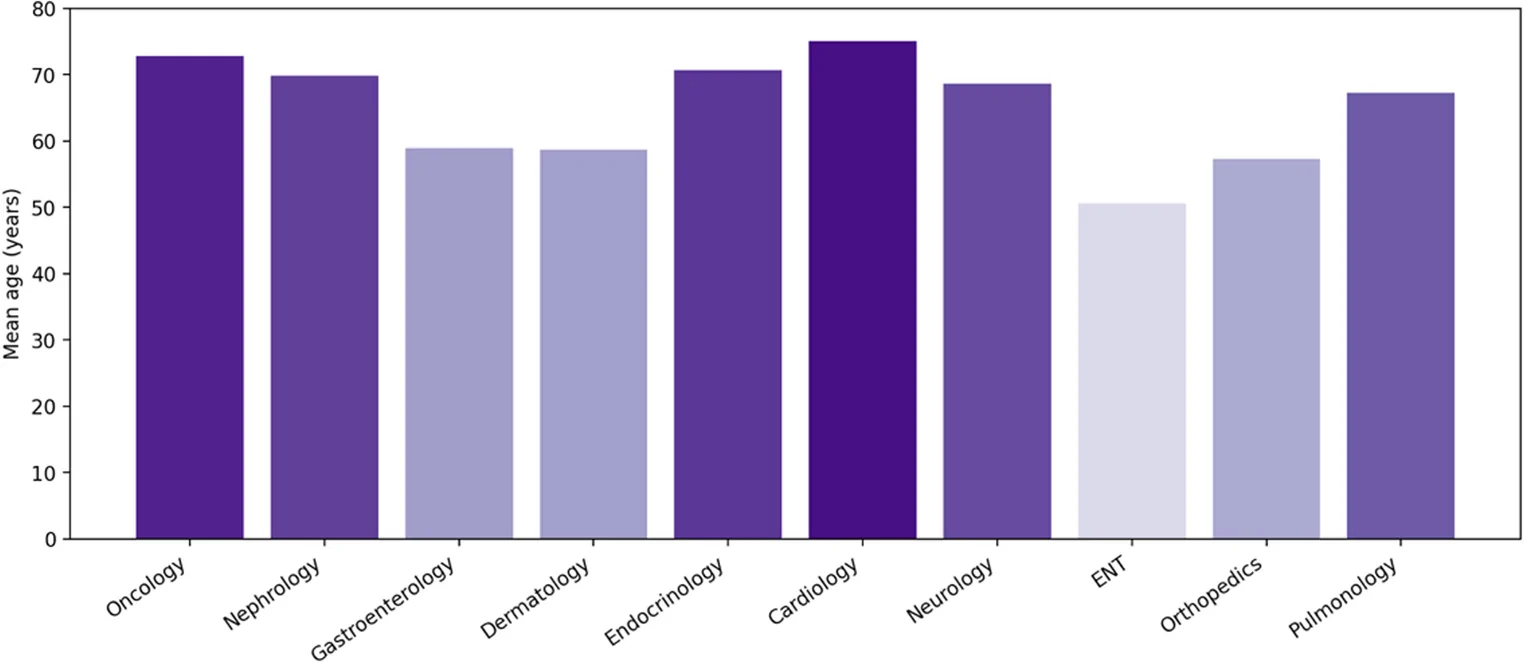
Mean age distribution across medical specialties in the study cohort. The bar chart displays the mean patient age (in years) for 10 medical specialties. The specialties shown include oncology, nephrology, gastroenterology, dermatology, endocrinology, cardiology, neurology, ENT (ear, nose, and throat), orthopedics, and pulmonology. Mean ages range from approximately 50 years (ENT) to 75 years (Cardiology), reflecting the typical age profiles associated with different disease conditions

We utilized electronic health record (EHR) data from Froedtert Hospital in Wisconsin. We selected 30 common healthcare categories as subspecialties (e.g., Ischemic & Coronary Disease, Epilepsy & Headache, Diabetes & Metabolic Disorders). For each subspecialty, we randomly sampled 2,500 unique patients who had at least one relevant diagnosis code and extracted their complete medical history, yielding a total cohort of 75,000 patients. For each patient, we extracted demographics, diagnosis history, procedures, laboratory tests and results, and medications. Figure 2 shows the mean age distribution across the 10 medical specialties in our cohort.

Demographic analysis revealed typical patterns for a tertiary care hospital population. The cohort was 56.2% female and 43.8% male, with consistent gender distribution across specialties. The most prevalent diagnoses included essential hypertension (I10), unspecified hyperlipidemia (E78.5), and type 2 diabetes mellitus without complications (E11.9). Our cohort’s mean age was 64.99 years, however the specialty breakdown showed cardiology patients averaged 74.96 years which was the highest average among all specialties, reflecting the age-related nature of cardiovascular disease, while ENT patients averaged 50.55 years, the lowest amongst the cohort. The Full cohort is included in Supplementary Table 1 (Online Resource 1).

#### Temporal Sequence Construction

**Fig. 3:**
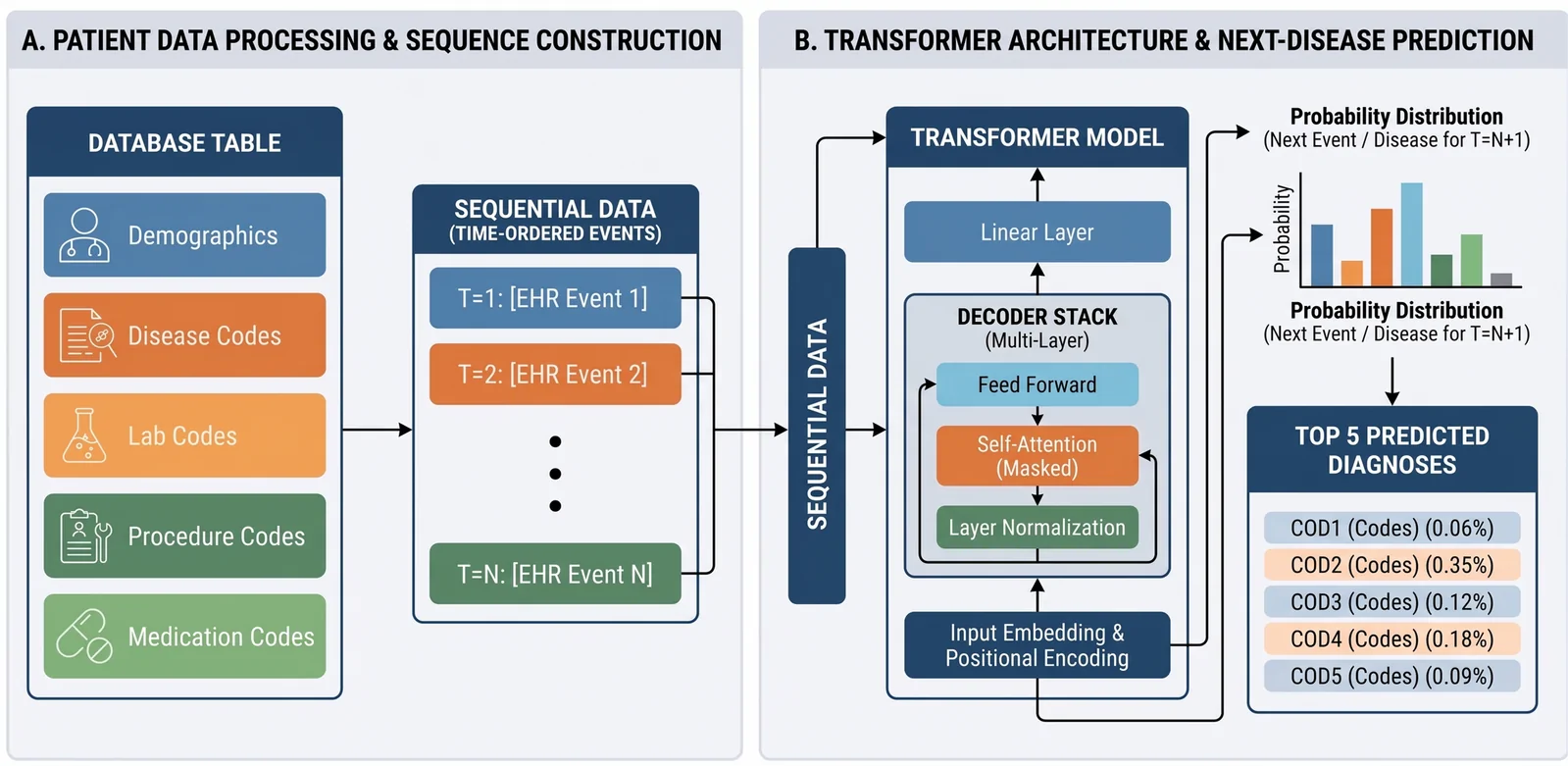
Data processing and transformer architecture pipeline. Structured EHR data (demographics, disease codes, laboratory codes, procedure codes, medication codes) are transformed into sequential representations with timestamped tokens. The transformer processes embedded sequences through multiple decoder layers to generate probability distributions over candidate diagnoses

To prepare the data for sequential modeling, we transformed the tabular data into longitudinal sequences of clinical events. Each event was converted into a discrete token that encoded both its type (e.g., diagnosis, procedure, medication) and its specific medical code. Events were ordered chronologically based on when they occurred in the patient’s medical history. Each token was paired with a corresponding time index representing the number of days elapsed since the patient’s first recorded event. Figure 3 illustrates the complete data processing and modeling pipeline from the structured database table through sequential representation to transformer-based prediction.

We augmented each patient sequence with a start token at time zero and an end token at the final recorded event, enabling teacher forcing during training. To reduce redundancy, we removed consecutive duplicate codes, retaining only the first occurrence in any uninterrupted series of identical events. Patient sequences exhibited variability in length, with a mean of 1,881 events per patient (standard deviation: 3,137) and a median of 725 events (Online Resource 1). Nephrology patients had the longest sequences, averaging 2,370 events. In contrast, ENT patients averaged 1,502 events, aligning with both their younger mean age and the typically episodic nature of otolaryngologic conditions. Full sequence length and mean age by specialties are reported in Supplementary Table 2 (Online Resource 1).

#### Hierarchical Dataset

We structured the data into three hierarchical levels to train models for different stages of the clinical diagnostic process. At the highest level, we created a single primary care dataset that included all 72,000 patients. For this dataset, all diagnosis codes were mapped to their general specialty (e.g., ‘Cardiology’, ‘Neurology’). This abstraction enabled the training of a high-level triage model designed to predict the most probable specialty referral based on a patient’s longitudinal history.

At the intermediate level, we established 10 specialty datasets by aggregating patients from their corresponding subspecialties. Each specialty encompassed 3 or more subspecialties. Within these datasets, we mapped specific diagnosis codes to broad, clinically coherent categories. For instance, in the cardiology dataset, diverse arrhythmia codes such as atrial fibrillation, ventricular tachycardia, and heart block were consolidated into a single arrhythmia category.

At the most granular level, we created 30 subspecialty-specific datasets. Each dataset, comprising 2,400 patients, contained the complete, uncategorized sequences of medical codes (e.g., ICD-10, LOINC, CPT). These datasets were used to train transformer models for the task of predicting the exact next diagnosis code. The specific ICD-10 target codes assigned to each subspecialty are provided in Supplementary Table 3 (Online Resource 1).

### Predictive Transformer Models

To equip the agents in our framework with data-driven predictive capabilities, we developed and trained a suite of 41 sequential transformer models (30 subspecialties, 10 specialties and 1 primary care). All models were built upon a shared architecture and trained with identical hyperparameters, with variations only in their specific vocabularies and training datasets.

#### Tokenization

For each of the 41 datasets, we constructed a unique vocabulary containing all event tokens observed in the training data. The vocabulary size and composition for each model are provided in Supplementary Table 4 (Online Resource 1). This vocabulary was augmented with four special tokens to denote padding, sequence start, sequence end, and unknown events (e.g. PAD, EOS, SOS, UNK). To make the processing of long sequences computationally feasible, we employed a sliding window approach. Patient histories were segmented into windows of 64 tokens, with an overlap of 4 tokens between adjacent windows. Within each window, the model’s objective was to predict the final token based on the preceding 63 tokens. Tokenization and input construction parameters are provided in Supplementary Table 5 (Online Resource 1).

#### Embeddings

To create meaningful representations for our transformer model, we encoded each token using three distinct embedding components. For a token ***x*** at position ***i*** in a sequence, with event type ***type(x)*** and relative time index ***time(x)***, the initial embedding was formulated as:

$$\:{E}_{initial}\left({x}_{i}\right)={E}_{token}\left({x}_{i}\right)+{E}_{type}\left(type\left({x}_{i}\right)\right)+{E}_{time}\left(time\left({x}_{i}\right)\right)$$

***E_token***, ***E_type***, and ***E_time*** are learnable embedding matrices, each with a hidden dimension of 512. This approach allows the model to simultaneously capture what happened (the specific medical code), what kind of event it was (diagnosis versus procedure versus medication), and when it occurred relative to the patient’s medical history.

For diagnosis tokens, we further enriched this representation by incorporating information from their hierarchical ancestors in the ICD coding system. Let ***A(xi)*** represent the set of ancestor codes for a diagnosis token ***xi***. The final input embedding was computed as:

$$\:{E}_{final}\left({x}_{i}\right)={E}_{initial}\left({x}_{i}\right)+\alpha\:\cdot\:\frac{1}{\left|A\left({x}_{i}\right)\right|}\cdot\:{\sum\:}_{a\in\:A\left({x}_{i}\right)}{E}_{token}\left(a\right)$$

where ***α*** is a learnable scaling factor that controls the influence of ancestor information. This formulation enables the model to understand that a specific diagnosis is not only its own distinct condition but also inherits properties from broader categories. We also applied a dropout layer with probability 0.2 to the final embedding for regularization. The complete embedding configuration is provided in Supplementary Table 6 (Online Resource 1).

#### Transformer Architecture

We implemented a decoder-only transformer architecture to predict the next-event in a patient’s sequence. The architecture consisted of a stack of four transformer layers. Each layer contained a causal multi-head self-attention mechanism with eight parallel attention heads and a position-wise feed-forward network. To preserve the autoregressive property required for sequence generation, causal masking was applied within the attention mechanism, preventing any position from attending to subsequent positions. The output of the attention sub-layer was passed to a feed-forward network, which consisted of two linear transformations with a gated activation function, SwiGLU, to enhance expressivity. Full architecture hyperparameters are provided in Supplementary Table 7 (Online Resource 1).

#### Model Training

The models were trained on a next-token prediction objective. The cross-entropy loss was calculated only for non-padding tokens in the sequence. To improve generalization, we incorporated label smoothing with a smoothing factor of 0.1. We configured the AdamW optimizer with a learning rate of 1 × 10⁻⁴ and a weight decay of 0.1. To prevent exploding gradients during training, we applied gradient clipping with a maximum global norm of 1.0. Each model was trained for a maximum of 500 epochs with an early stopping protocol based on validation loss, with a patience of 10 epochs. For each of the 41 datasets, we partitioned patients into training (95%), validation (5%) on 72,000 patient train set, and 3000 patients were excluded separately for the test set. The full training setup is provided in Supplementary Table 8 (Online Resource 1).

### Multi-Agent Clinical Framework

#### Agent Roles and Hierarchical Structure

**Fig. 4:**
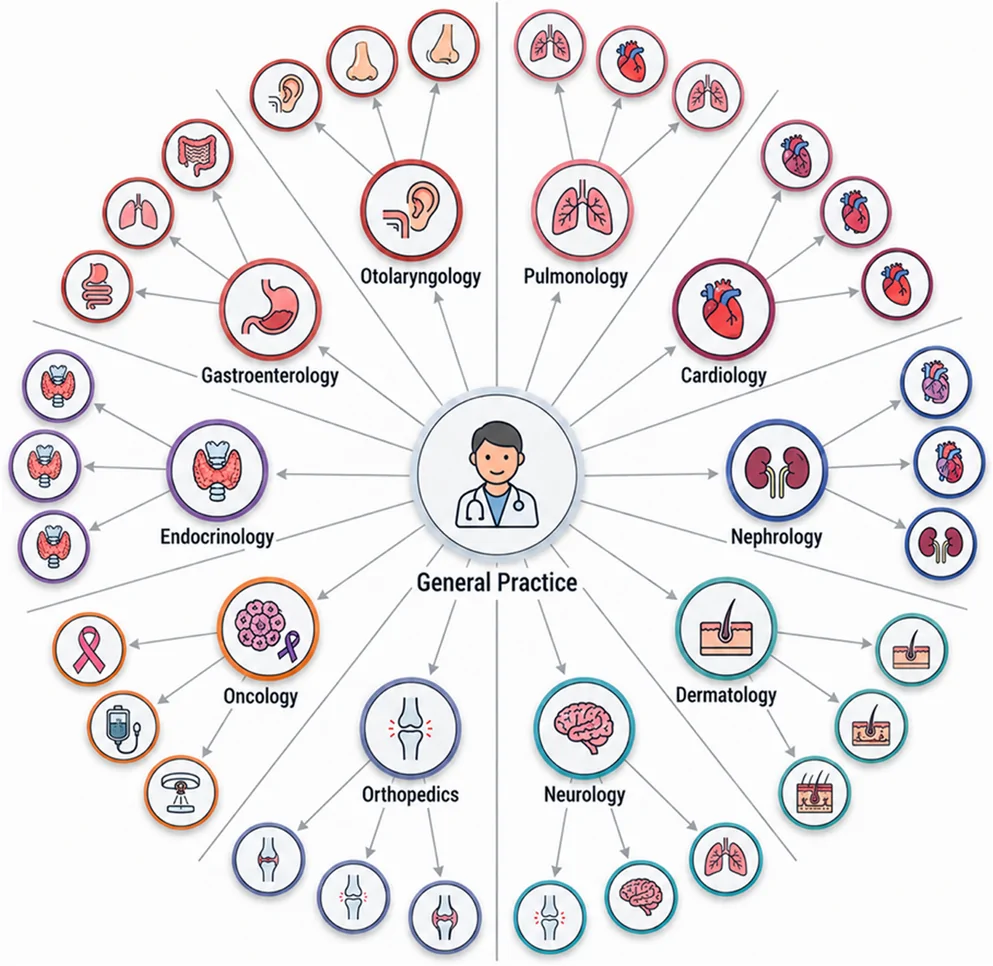
Agentic graph framework architecture. The primary care agent (center) receives patient cases and refers to one of 10 specialty agents. Each specialty agent connects to 3 subspecialist agents, with each subspecialist focusing on specific disease categories within their domain. Arrows indicate possible referral pathways through the decentralized consultation network

To simulate the current diagnostic framework in clinical settings, we designed CPS-Net so that each agent in the framework can function as a simulated physician, with its reasoning powered by a single, deterministic large language model (DeepSeek-flash). The agents are not fine-tuned and receive no specialty-specific training data. All agents share the same base model and rely on the medical knowledge contained in the standard DeepSeek pretraining corpus, with their behavior differentiated only by assigned role, system prompt, and the transformer each may query. Specialty-specific prediction is therefore supplied by the transformers rather than by any per-agent training. Agent judgment is likewise bounded rather than open-ended: each agent is shown the transformer’s ranked codes together with their probabilities and is instructed to retain the top-ranked code by default, departing from it only under two defined conditions. The first is when the record documents an active, progressing problem in a different organ system, which prompts a cross-specialty redirection. The second is when a lower-ranked code is the exact variant the record uses, which prompts a within-subspecialty reordering. Absent either condition, the transformer’s ranking stands. Figure 4 illustrates the graph structure of the framework across primary care, specialty, and subspecialty tiers.

The **Primary Care** Agent serves as the initial point of contact for all patient cases. This agent processes the patient’s complete medical history as a sequential record and generates a clinical summary in natural language. It is equipped with a broadly trained transformer that provides predictions for the most likely specialty category of the next diagnosis. The agent uses this probabilistic guidance to refer the case to an appropriate specialty agent. The agent integrates this probabilistic guidance with its own clinical reasoning, derived from large language model training on general medical knowledge, to re-rank the model’s output if needed and refer the case to an appropriate specialty agent.

The second tier consists of 10 **Specialty Agents**, each corresponding to a major medical specialty. Upon receiving a referral, a specialty agent assesses the case using the provided clinical summary and the patient’s structured history. It queries a specialty-level transformer, which predicts the most probable subspecialty disease groups. Based on this analysis, the specialty agent either escalates the case to one of its corresponding subspecialist agents or, if the evidence suggests a different underlying condition, redirects the referral to another specialty.

The third and most specialized tier comprises 30 **Subspecialty Agents**. Each of these agents has exclusive access to a dedicated subspecialty transformer. When a case is referred to a subspecialist, the agent accesses the complete conversation history and patient data. It then utilizes its transformer to generate top-10 predictions for the next specific diagnosis code, along with their associated probabilities. The agent synthesizes these predictions with the clinical context to formulate a final diagnosis or a ranked different diagnosis.

All agents and their interactions are managed within a stateful computation graph implemented using LangGraph. The state of the graph maintains the patient journey, pre-computed transformer inputs, the conversation history, and other relevant metadata. The process progresses through the graph based on the agents’ routing decisions until a concluding diagnosis is reached.

#### Collaboration Protocol

The framework operates on a decentralized, orchestrator-free communication protocol that allows agents to interact directly through peer consultation. Communication occurs through structured messages containing both a clinical narrative and a decision layer. The narrative synthesizes an updated assessment of the patient’s condition with the cumulative case history, while the decision layer specifies the next action (such as referring to another agent or finalizing a diagnosis) along with explicit reasoning. They can figure out which agent raised what concerns, what evidence supported each referral (e.g. other agents’ transformer output), which specialists considered and ruled out alternative diagnoses, and where uncertainties emerged.

To ensure the quality of the collaborative process, the framework enforces a few rules. First, an agent must contribute new clinical information or reasoning before making a referral. Second, the system tracks the consultation history to prevent redundant or circular referrals. Third, all referrals must be explicit, naming the target agent and providing a clear clinical rationale. A case concludes when a subspecialty agent issues a final answer, which includes the primary diagnosis, a ranked list of differential diagnoses, and a concise explanation that links the conclusion to the patient’s history and the preceding agent-based reasoning.

#### Evaluation Protocol

For evaluation, we constructed a test set by randomly sampling 100 different patients from each of the 30 subspecialties, yielding 3,000 cases. For each case, we truncated the patient’s longitudinal history just before the latest occurrence of their assigned subspecialty’s key disease codes, designating that code as the prediction target. We employed a dual evaluation strategy to capture both technical precision and clinical utility. Two researchers independently evaluated the system’s predictions for each case. We quantified performance using top-k accuracy (k = 1, 3, and 5) based on exact ICD-10 code matching, reflecting the system’s ability to retrieve the precise diagnosis. We also assigned binary clinical relevance labels for each prediction. A prediction was deemed clinically relevant if it represented the same clinically condition within the correct specialty.

### Baseline Models for Comparison

To contextualize the performance of our CPS-Net, we developed and evaluated four baseline models: (1) a single LLM agent without specialized transformers or multi-agent structure, (2) a single monolithic transformer trained on the entire dataset to assess the value of specialty-specific training, (3) a centrally orchestrated multi-agent system to evaluate the impact of our decentralized communication protocol, and (4) a hierarchical transformer baseline that shares CPS-Net’s models but omits the consultation protocol, isolating the contribution of specialty decomposition from that of multi-clinic consultation and re-ranking.

#### Single Large Language Model Agent Baseline

This baseline was designed to assess the diagnostic capability of a large language model in isolation, without specialized transformers or a multi-agent structure. We used the same base LLM (DeepSeek-flash) as in our framework. For each of the 3,000 test cases, the model was provided with the patient’s truncated medical history and a standardized prompt to predict the most likely next diagnosis. The model’s responses, including the primary diagnosis and any listed alternatives, were evaluated using the same manual review process as the main framework (top-k accuracy and clinical relevance).

#### Single Monolithic Transformer Baseline

To investigate the value of our specialty-specific training strategy, we trained a single, large-scale monolithic transformer. This model was trained on the entire 72,000-patient dataset, with a vocabulary encompassing 22,000 + unique exact medical codes. We evaluated this model on the same test cases, using its next-token predictions to assess top-k accuracy. This baseline allowed us to directly compare the performance of a specialized, decomposed modeling approach against a single, high-capacity generalist model.

#### Orchestrated Multi-Agent Baseline

To specifically evaluate the impact of our decentralized communication protocol, we implemented a centrally orchestrated version of the multi-agent system. This baseline introduced a supervisor agent that controlled the flow of consultation. After each agent’s turn, the supervisor reviewed the conversation and decided which agent should act next. The individual specialist agents retained their roles and access to their respective transformers, but all routing decisions were centralized. We evaluated this orchestrated system on the same cases, using the same metrics and transformers.

#### Hierarchical Transformers

To isolate the contribution of multi-clinic consultation and agent-based re-ranking from that of specialty decomposition alone, we evaluated a hierarchical transformer baseline that shares CPS-Net’s models but not its consultation protocol. This baseline used the identical two-tier routing chain, in which a primary care model predicts the specialty of the next diagnosis and the corresponding specialty model then selects among its subspecialties, together with the identical 30 subspecialty transformers. At each hop the learned transformer model proposed its five most probable destinations, after which the case was committed to a single subspecialty. That subspecialty transformer’s top-5 next-diagnosis predictions were then returned as the final answer, with no consultation of alternative clinics and no re-ranking of candidate codes.

## Results

### Next-Disease Prediction

To evaluate our framework’s diagnostic performance, we assessed next disease prediction against our gold standard dataset. Table 1 presents the core performance metrics. CPS-Net achieved top-1, top-3, and top-5 accuracies of 48.8%, 71.00%, and 77.76%, respectively. Compared with the monolithic transformer, our framework showed consistent gains across all metrics, with improvements of 29.6% points in top-1 accuracy, 32.40% points in top-3 accuracy, and 30.56% points in top-5 accuracy.

**Table 1:** Overall next-disease prediction performance across pipelines

Model / Pipeline	Top-1 (%)	Top-3 (%)	Top-5 (%)	Relevance (%)
Single Large Language Model Agent	12.7	26.40	37.23	68.73
Monolithic transformer	19.2	38.60	47.20	73.81
CPS-Net	**48.8**	**71.00**	**77.76**	**94.17**
Hierarchical transformers	44.2	52.23	52.67	79.90

The single LLM agent performed poorly across all accuracy metrics despite achieving reasonable clinical relevance scores, revealing a distinction between conceptual medical knowledge and structured code prediction. LLMs are trained primarily on textual medical literature that associates diseases with signs and symptoms, rather than on longitudinal EHR sequences that capture disease progression patterns. These findings confirm that decomposing the prediction task according to medical specialization hierarchies, combined with training on structured longitudinal data, substantially improves predictive accuracy relative to general-purpose language models and undifferentiated monolithic transformer.

To isolate the effect of agent consultation from the underlying transformers, we compared CPS-Net with the hierarchical transformer baseline, which uses the same 41 models but returns each subspecialty transformer’s predictions without consultation or re-ranking. The baseline reached top-1, top-3, and top-5 accuracies of 44.2%, 52.23%, and 52.67%, while CPS-Net reached 48.8%, 71.00%, and 77.76%, corresponding to gains of 4.60, 18.77, and 25.10% points. Across the 3,000 test cases, the agents intervened in 1,011 cases (33.70%): at the primary care stage, redirecting to a different specialty, in 179 cases (5.97% of all cases); at the specialty stage, redirecting to a different subspecialty, in 55 cases (1.83% of all cases); and at the subspecialty stage, re-ranking the diagnosis codes, in 777 cases (25.90% of all cases). Redirection typically followed an active, progressing problem in an organ system outside the routed one, while re-ranking typically followed a lower-ranked code matching the variant the record used. Across all 1,011 interventions, the combined effect of redirection and re-ranking at top-1 was to promote the correct code into the retained prediction in 222 cases (7.40% of all cases) and to displace it in 84 cases (2.80%), a net improvement of 138 cases (4.60%). The same pattern widened at top-3, with 642 cases improved (21.40%) against 79 impaired (2.63%) for a net of 563 cases (18.77%), and at top-5, with 791 improved (26.37%) against 38 impaired (1.27%) for a net of 753 cases (25.10%). As k increased, beneficial interventions rose and harmful ones fell, indicating that the agents corrected more transformer errors than they introduced.

### Performance Variation Across Specialties and Subspecialties

We analyzed the framework’s performance across clinical domains to understand how effectiveness varied by specialty and subspecialty. Table 2 presents the best performing subspecialties for the single LLM agent and CPS-Net approaches.

**Table 2:** Subspecialty performance highlights

Pipeline	Best Top-5 subspecialty	Top-5 (%)
A. Single LLM	Endocrinology–Diabetes and Metabolic	65.00
B. Monolithic transformer	Orthopedics–Pediatric and Congenital	64.00
C. CPS-Net	**Oncology–Hematology**	**95.00**
D. Hierarchical (no LLM)	Oncology–Hematology	88.00

For CPS-Net, Oncology–Hematology achieved the highest top-5 accuracy at 95.00%. The single LLM agent’s best top-5 performance reached 65.00%, in Endocrinology–Diabetes and Metabolic.

### Qualitative Evaluation of Multi-Agent Consultations

**Fig. 5:**
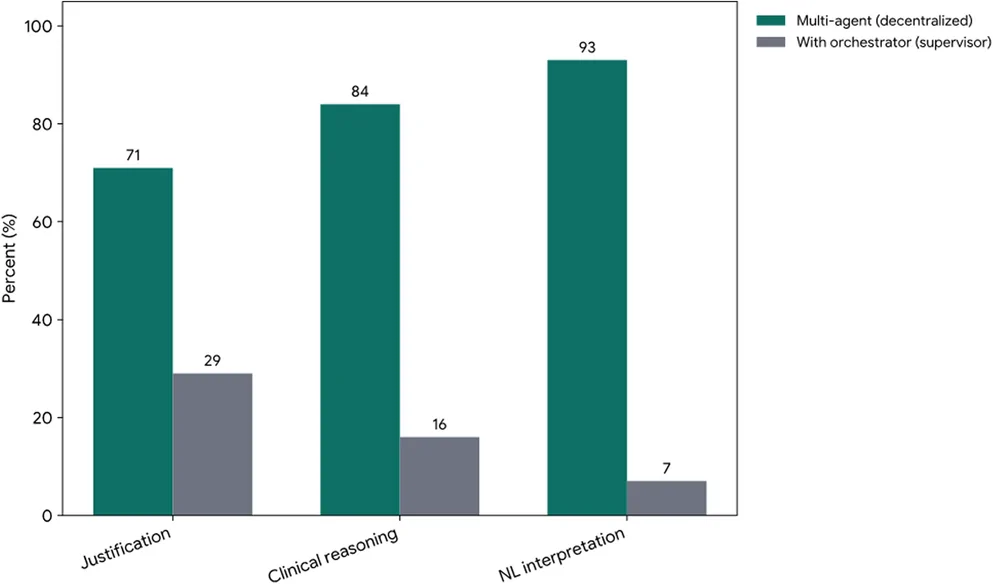
Comparison of CPS-Net versus supervisor-based multi-agent architectures. The decentralized framework was preferred over the orchestrator-based variant by an independent language model judge for justification quality (71% vs. 29%), clinical reasoning coherence (84% vs. 16%), and natural language interpretation (93% vs. 7%)

To validate our decentralized architectural design, we compared the framework to a baseline system that used a central supervisor agent to orchestrate consultation flow. Figure 5 presents the qualitative evaluation results.

We conducted a qualitative evaluation using a separate language model (GPT o1) as an impartial judge to compare reasoning quality. For 100 randomly selected cases, the judge was presented with conversation transcripts from both frameworks in a blinded manner and asked to choose the superior approach across three criteria: justification quality, clinical reasoning coherence, and natural language interpretation of predictions. The judge strongly preferred the decentralized framework across all dimensions: justification (71%), clinical reasoning (84%), and natural language interpretation (93%). The direct agent-to-agent communication resulted in more transparent decision-making processes that were easier to follow and audit. Beyond these gains, we quantified information preservation during agent handoffs. In the supervisor-based architecture, an average of 8% of information was lost during routing through the central orchestrator, as the supervisor filtered messages between agents.

### Incorrect Predictions

We analyzed the framework’s incorrect predictions to assess whether they might represent clinically meaningful risk signals rather than true errors. For cases where the model’s predictions did not match the immediate next diagnosis in the patient’s record, we reviewed the patient’s complete untruncated medical history to determine whether any of the predicted diagnoses appeared subsequently. We defined the look-ahead window as the entire remaining patient history following the truncation point.

Among top-1 predictions that were incorrect for the immediate next diagnosis, 53.08% of the predicted codes appeared later in the patient’s history. For top-3 predictions, 71.82% included at least one code that subsequently occurred, and for top-5 predictions, the rate reached 76.16%. These findings reveal that a substantial majority of predictions initially classified as errors actually corresponded to diagnoses the patient eventually received. This pattern suggests the framework captures latent disease trajectories and emerging risk patterns that manifest over extended timeframes rather than merely predicting the immediate next event.

## Discussion

### Principal Findings

We designed an agent swarm that simulates hospital diagnostic workflows through hierarchical specialty-based consultation. By structuring the system into primary care, specialty, and subspecialty tiers, each connected to focused transformers trained on progressively narrower disease domains, we demonstrated that architectural alignment with clinical practice improves both predictive performance and interpretability. The orchestrator-free design enabled direct agent collaboration without information loss, addressing a fundamental limitation of centrally mediated multi-agent systems.

Our results establish three key findings. First, hierarchical task decomposition improves prediction accuracy compared to monolithic approaches. CPS-Net achieved accuracy of 48.8%, 71.00%, and 77.76% in top-1, top-3, and top-5 next-disease prediction, respectively, outperforming a single transformer trained on all patients. Second, structured collaboration grounded in longitudinal EHR data substantially outperforms general-purpose language models. Using a single DeepSeek-flash achieved only 12.70%, 26.40%, and 37.23% accuracy in top-1, top-3, and top-5 predictions despite reaching 68.73% clinical relevance. This finding demonstrates that accuracy gains in our framework arise from architectural design and training on structured longitudinal data, not from language generation capabilities alone. Third, removing the central supervisor enhances reasoning transparency. Qualitative evaluation by an independent language model judge showed the decentralized system was strongly preferred for justification quality (71%), clinical reasoning coherence (84%), and natural language interpretation (93%). These improvements show the supervisor’s filtering and reformulation of messages created bottlenecks that degraded both the richness of clinical context and the transparency of decision logic. In contrast, the decentralized architecture made the complete reasoning chain auditable.

### Implications

This framework represents a paradigm for clinical decision support. Physicians can review structured conversation histories that document the complete diagnostic trajectory, including which specialists were consulted, what evidence informed each referral decision, and which alternative diagnoses were considered and excluded. The framework’s capability to capture latent disease trajectories suggests potential applications in prospective risk stratification. When the system predicts disease codes that do not match the immediate presentation but appear in specialized domains, these signals could trigger automated alerts for clinician review. The hierarchical consultation process may enhance diagnostic accuracy by simulating the collaborative deliberation that occurs in multidisciplinary case conferences. Specialist agents can surface domain-specific insights, while the transparent communication protocol ensures that concerns raised by any agent remain visible throughout the diagnostic process. This architecture provides a framework for augmenting rather than replacing clinical judgment, offering physicians structured decision support.

### Prior Work

Early multi-agent clinical decision support emphasized on limited number of disease prediction. González-Vélez et al. introduced HealthAgents [34], an agent-based, multi-center decision support system for brain tumor diagnosis and prognosis, designed to securely connect clinical sites and coordinate analysis across the network. For cardiovascular screening, Elhoseny et al. proposed a multi-agent feature wrapper (MAFW) [35] that splits feature selection into interacting tasks: preparation, selection, classification, and evaluation. On the UCI Cleveland heart disease dataset, their hybrid model reached 90.1% accuracy with 88.9% precision and 98.4% recall.

More recently, LLM-based multi-agent consultation frameworks have emerged to mimic specialist deliberation directly. Chen et al. developed a Multi-Agent Conversation (MAC) [33] framework and evaluated it on 302 rare-disease case reports. With GPT-4 and four doctor agents, MAC achieved 34.11% accuracy for the most likely diagnosis and improved recommended-test helpfulness to 78.26% in primary consultation. Wang et al. introduced AMSC [36], which fuses specialist-agent probability distributions via a self-attention APDF module. By freezing the LLM and training only APDF, AMSC reached 86.1% accuracy on the DXY dataset and reported up to 3.2% accuracy gains with 95.5% training-time savings. Although these studies advanced multi-agent frameworks for diagnosis, they share critical limitations. First, no agents are trained on longitudinal EHR data, leaving a fundamental gap in understanding disease progression patterns, structured medical coding systems, and clinical context. Second, most studies did not address whether multi-agent reasoning remains auditable and evidence-grounded when agents freely discuss and share information beyond controlled case narratives. Real-world clinical data contains incomplete information, coding variability, and temporal complexity that may challenge the transparency and reliability of LLM-based consultation.

### Case Example

**Fig. 6:**
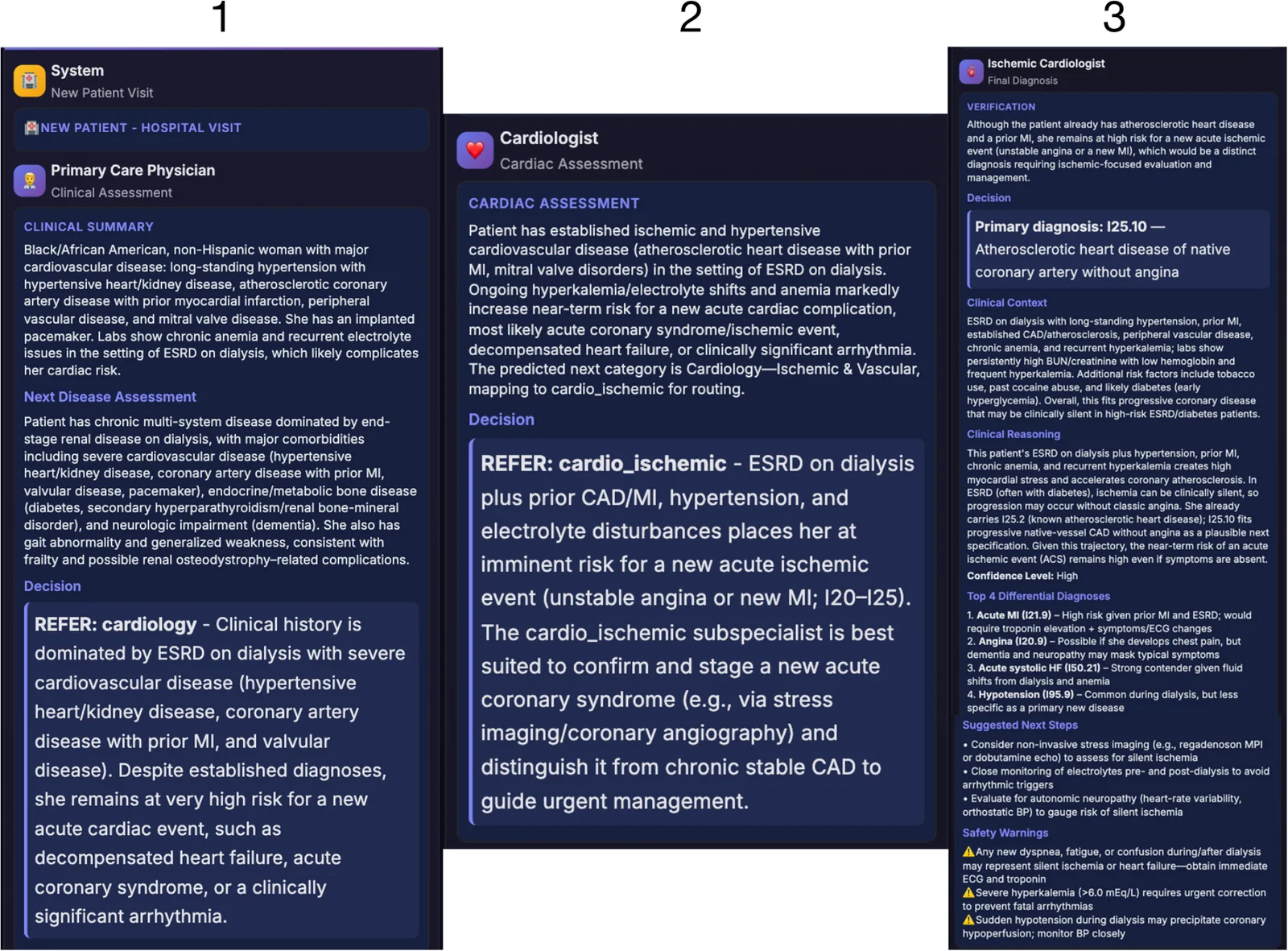
Illustrative case demonstrating CPS-Net consultation workflow. Panel **1** shows the primary care agent’s clinical summary and referral decision to cardiology based on the patient’s cardiovascular risk profile. Panel **2** displays the cardiology specialist’s assessment and subsequent referral to the ischemic cardiology subspecialist. Panel **3** presents the subspecialist’s final diagnosis (I25.10), clinical reasoning, differential diagnoses, and recommended next steps. The transparent consultation chain allows clinicians to audit each decision point and understand the evidence supporting the diagnostic conclusion

To demonstrate how the framework operates, we present a representative case: a 67-year-old female with end-stage renal disease on dialysis, prior myocardial infarction, atherosclerotic heart disease, and multiple comorbidities. Her longitudinal record contains 3,580 clinical events. The prediction target is I25.10, atherosclerotic heart disease without angina. Figure 6 illustrates the complete consultation workflow across the three hierarchical tiers.

The primary care agent receives the complete structured history and generates a clinical summary synthesizing her complex trajectory. The primary care transformer predicts cardiology (24.5% probability), followed by endocrinology and orthopedics. The agent reviews these predictions and determines that given her progressive cardiovascular and renal disease, the highest risk is a new acute cardiac event. It refers to cardiology with explicit reasoning.

The cardiology agent assesses her cardiovascular risk profile and notes she remains at extremely high risk for new events despite existing cardiac diagnoses. The cardiology transformer predicts ischemic and vascular disease as the most probable category. The agent refers to the cardio_ischemic subspecialist, explaining the risk for acute coronary syndrome distinct from chronic stable disease.

The subspecialist’s transformer generates top-5 predictions: I25.2 (38.7%), I25.10 (34.6%), I50.20 (2.7%), I50.22 (1.2%), and I95.9 (1.1%). The agent selects I25.10 as primary diagnosis, explaining this represents progressive silent coronary disease likely advancing without typical symptoms due to autonomic neuropathy. It also provides clinical reasoning, differential diagnoses, suggested diagnostic steps, and safety warnings before referring back to primary care.

### Limitations

The framework was developed on data from a single tertiary care hospital, which might limit its generalizability. Moreover, the framework could be evaluated and analyzed by specialist medical physicians in order to assess whether the reasoning patterns and referral decisions align with actual clinical practice and identify domain-specific errors that quantitative metrics might miss. The evaluation used retrospective EHR data and relied on coded diagnoses as ground truth rather than clinician-confirmed diagnostic intent, which may not accurately reflect clinical reasoning at the time of documentation.

### Future Directions

Prospective validation in multiple clinical sites is needed. The framework should be tested with real clinicians reviewing predictions and conversation traces to assess whether it provides practical value in actual care settings. Integration of imaging summaries, genome sequencing, and clinical notes may improve prediction accuracy. Agent roles could be updated dynamically as new evidence emerges during patient care, allowing the system to refine its reasoning when additional information becomes available. The framework could learn from clinician feedback to refine referral decisions over time. Human-in-the-loop evaluation with practicing physicians is necessary to assess usability and trust.

## Conclusion

This study introduced a decomposing diagnostic support framework that aligns predictive modeling with how diagnosis actually unfolds in clinical practice. By coupling 41 trained transformers with a decentralized multi-agent consultation layer, the system makes the diagnostic path interpretable through transparent referral dialogue that clinicians can review. Across 3,000 test cases, the framework achieved top-1, top-3, and top-5 accuracies of 48.8%, 71.00%, and 77.76% with 94.17% clinical relevance, substantially outperforming both a monolithic transformer and a general-purpose language model. Notably, many incorrect predictions appeared later in patient records, suggesting the framework captures disease trajectories beyond immediate events.

These findings support a central implication: architectures that mirror clinical specialization hierarchies improve both accuracy and interpretability for diagnostic prediction from longitudinal electronic health records. The framework provides a foundation for decision support tools that reflect how physicians collaborate while making reasoning transparent for validation. Future work should focus on multi-site prospective validation, clinician evaluation of referral logic, integration of multimodal data including clinical notes and imaging, and human-in-the-loop feedback mechanisms to refine referral decisions and maintain real-world trustworthiness.

## Supplementary Information

Below is the link to the electronic supplementary material.

Supplementary Material 1

## Data Availability

The de-identified patient data used in this study were accessed through the Froedtert research network. Restrictions apply to the availability of these data, which were used under license for the current study and are therefore not publicly available. Information on accessing the platform is available on the Froedtert website. The de-identified patient data for external validation used in this study were sourced from patient records from Froedtert & the Medical College of Wisconsin health system and cannot be shared publicly due to confidentiality agreements and ethical restrictions. However, summary data and statistical analysis results are available upon reasonable request to the corresponding author.
