# Supplementary material for: CPS-Net (Collaborating Physicians in Silico Network): A Decentralized Multi-Agent Transformer Framework for Specialty-Aware Disease Prediction
Source: J Med Syst. 2026 Sep 15;50(1):131. doi: 10.1007/s10916-026-02460-8 (PMC13578076; doi:10.1007/s10916-026-02460-8)
Supplement: Supplementary file 1 [file 10916_2026_2460_MOESM1_ESM.docx]

**Supplementary Materials**

Supplementary Table 1 - Full Cohort Table

| **Metric** | **Value** |
| --- | --- |
| Total Number of Patients | 75000 |
| **Age** |  |
| Average | 64.99 |
| Standard Deviation | 21.70 |
| Median | 68.85 |
| **Gender** |  |
| Male% | 43.79 |
| Female% | 56.20 |
| **Race** |  |
| White | 79.59 |
| Black | 13.62 |
| Asian | 1.63 |
| Other | 5.13 |
| **Ethnicity** |  |
| Hispanic | 3.67 |
| Non Hispanic | 94.86 |
| **Sequence Length** |  |
| Mean | 1881.31 |
| Median | 725.0 |

Supplementary Table 2 – Mean Age and Sequence Length for specialties

| **Specialty** | **Sequence Length Mean (SD)** | **Age Mean** |
| --- | --- | --- |
| Nephrology | 2,370 (3,480) | 69.8 |
| Pulmonology | 2,358 (3,509) | 67.2 |
| Cardiology | 2,232 (3,439) | 75.0 |
| Oncology | 2,100 (3,047) | 72.8 |
| Gastroenterology | 1,880 (3,146) | 58.9 |
| Orthopedics | 1,666 (2,891) | 57.3 |
| Dermatology | 1,594 (2,836) | 58.7 |
| Endocrinology | 1,560 (2,427) | 70.7 |
| Neurology | 1,538 (2,815) | 68.6 |
| ENT | 1,502 (2,875) | 50.6 |

Supplementary Table 3 – Code Mapping

| **Specialty** | **Subspecialty** | **ICD-10 Code** | **Description** |
| --- | --- | --- | --- |
| Cardiology | Arrhythmias & Electrophysiology | I48.91 | Unspecified atrial fibrillation |
| Cardiology | Arrhythmias & Electrophysiology | I47.10 | Supraventricular tachycardia unspecified |
| Cardiology | Arrhythmias & Electrophysiology | I49.9 | Cardiac arrhythmia unspecified |
| Cardiology | Heart Failure & Structural Disease | I50.9 | Heart failure unspecified |
| Cardiology | Heart Failure & Structural Disease | I34.0 | Nonrheumatic mitral (valve) insufficiency |
| Cardiology | Heart Failure & Structural Disease | Q21.1 | Atrial septal defect |
| Cardiology | Ischemic & Vascular Disease | I21.9 | Acute myocardial infarction unspecified |
| Cardiology | Ischemic & Vascular Disease | I25.10 | Atherosclerotic heart disease of native coronary artery, without angina pectoris |
| Cardiology | Ischemic & Vascular Disease | I20.9 | Angina pectoris unspecified |
| Dermatology | Inflammatory & Autoimmune Skin | L20.9 | Atopic dermatitis unspecified |
| Dermatology | Inflammatory & Autoimmune Skin | L40.9 | Psoriasis unspecified |
| Dermatology | Inflammatory & Autoimmune Skin | L50.9 | Urticaria unspecified |
| Dermatology | Skin Cancer & Precancer | C44.91 | Other and unspecified malignant neoplasm of skin, unspecified |
| Dermatology | Skin Cancer & Precancer | C43.9 | Malignant melanoma of skin unspecified |
| Dermatology | Skin Cancer & Precancer | D04.9 | Carcinoma in situ of skin unspecified |
| Dermatology | Hair & Nail | L65.9 | Nonscarring hair loss unspecified |
| Dermatology | Hair & Nail | L63.9 | Alopecia areata unspecified |
| Dermatology | Hair & Nail | L60.0 | Ingrowing nail |
| ENT | Ear & Balance | H66.90 | Otitis media unspecified unspecified ear |
| ENT | Ear & Balance | H60.9 | Otitis externa unspecified |
| ENT | Ear & Balance | H81.9 | Unspecified disorder of vestibular function |
| ENT | Nose & Sinus | J31.0 | Chronic rhinitis |
| ENT | Nose & Sinus | J32.9 | Chronic sinusitis unspecified |
| ENT | Nose & Sinus | J34.2 | Deviated nasal septum |
| ENT | Throat & Head-Neck | J38.00 | Paralysis of vocal cords and larynx, unspecified |
| ENT | Throat & Head-Neck | J35.9 | Chronic disease of tonsils and adenoids, unspecified |
| ENT | Throat & Head-Neck | C32.9 | Malignant neoplasm of larynx unspecified |
| Endocrinology | Diabetes & Metabolic | E11.9 | Type 2 diabetes mellitus without complications |
| Endocrinology | Diabetes & Metabolic | E10.9 | Type 1 diabetes mellitus without complications |
| Endocrinology | Diabetes & Metabolic | E78.5 | Hyperlipidemia unspecified |
| Endocrinology | Thyroid & Pituitary | E03.9 | Hypothyroidism unspecified |
| Endocrinology | Thyroid & Pituitary | E05.90 | Thyrotoxicosis unspecified without thyrotoxic crisis |
| Endocrinology | Thyroid & Pituitary | E23.0 | Hypopituitarism |
| Endocrinology | Adrenal & Bone Disorders | E27.40 | Unspecified adrenocortical insufficiency |
| Endocrinology | Adrenal & Bone Disorders | M81.0 | Age-related osteoporosis without current pathological fracture |
| Endocrinology | Adrenal & Bone Disorders | E20.9 | Hypoparathyroidism unspecified |
| Gastroenterology | Inflammatory & Autoimmune GI | K50.90 | Crohn's disease, unspecified, without complications |
| Gastroenterology | Inflammatory & Autoimmune GI | K51.90 | Ulcerative colitis, unspecified, without complications |
| Gastroenterology | Inflammatory & Autoimmune GI | K52.9 | Noninfective gastroenteritis and colitis unspecified |
| Gastroenterology | Liver & Hepatology | K74.60 | Unspecified cirrhosis of liver |
| Gastroenterology | Liver & Hepatology | K76.0 | Fatty (change of) liver not elsewhere classified |
| Gastroenterology | Liver & Hepatology | B18.2 | Chronic viral hepatitis C |
| Gastroenterology | Pancreatic & Functional GI | K85.90 | Acute pancreatitis without necrosis or infection, unspecified |
| Gastroenterology | Pancreatic & Functional GI | K21.9 | Gastro-esophageal reflux disease without esophagitis |
| Gastroenterology | Pancreatic & Functional GI | K59.00 | Constipation unspecified |
| Nephrology | Acute & Glomerular Disorders | N17.9 | Acute kidney failure unspecified |
| Nephrology | Acute & Glomerular Disorders | N00.9 | Acute nephritic syndrome with unspecified morphologic changes |
| Nephrology | Acute & Glomerular Disorders | N05.9 | Unspecified nephritic syndrome with unspecified morphologic changes |
| Nephrology | Chronic Kidney Disease & Hypertension | N18.9 | Chronic kidney disease unspecified |
| Nephrology | Chronic Kidney Disease & Hypertension | I12.9 | Hypertensive chronic kidney disease with stage 1-4 or unspecified CKD |
| Nephrology | Chronic Kidney Disease & Hypertension | N25.9 | Disorder resulting from impaired renal tubular function unspecified |
| Nephrology | Stones & Transplantation | N20.0 | Calculus of kidney |
| Nephrology | Stones & Transplantation | N20.1 | Calculus of ureter |
| Nephrology | Stones & Transplantation | T86.10 | Unspecified complication of kidney transplant |
| Neurology | Epilepsy & Headache | G40.909 | Epilepsy, unspecified, not intractable, without status epilepticus |
| Neurology | Epilepsy & Headache | G43.909 | Migraine, unspecified, not intractable, without status migrainosus |
| Neurology | Epilepsy & Headache | R56.9 | Unspecified convulsions |
| Neurology | Neurodegenerative & Demyelinating | G20.A1 | Parkinson's disease without dyskinesia without fluctuations |
| Neurology | Neurodegenerative & Demyelinating | G35 | Multiple sclerosis |
| Neurology | Neurodegenerative & Demyelinating | F02.80 | Dementia in other diseases classified elsewhere without behavioral disturbance |
| Neurology | Stroke & Vascular Neurology | I63.9 | Cerebral infarction unspecified |
| Neurology | Stroke & Vascular Neurology | I61.9 | Nontraumatic intracerebral hemorrhage unspecified |
| Neurology | Stroke & Vascular Neurology | G45.9 | Transient cerebral ischemic attack unspecified |
| Oncology | Solid Tumors | C18.9 | Malignant neoplasm of colon unspecified |
| Oncology | Solid Tumors | C50.919 | Malignant neoplasm of unspecified site of unspecified female breast |
| Oncology | Solid Tumors | C34.90 | Malignant neoplasm of unspecified part of unspecified bronchus or lung |
| Oncology | Hematology | C91.00 | Acute lymphoblastic leukemia not having achieved remission |
| Oncology | Hematology | C92.00 | Acute myeloblastic leukemia, not having achieved remission |
| Oncology | Hematology | C85.90 | Non-Hodgkin lymphoma unspecified unspecified site |
| Oncology | Oncology | C56.9 | Malignant neoplasm of unspecified ovary |
| Oncology | Oncology | C61 | Malignant neoplasm of prostate |
| Orthopedics | Joint Disorders | M54.5 | Low back pain |
| Orthopedics | Joint Disorders | M16.9 | Osteoarthritis of hip unspecified |
| Orthopedics | Joint Disorders | M17.9 | Osteoarthritis of knee unspecified |
| Orthopedics | Trauma & Sports Injuries | S72.001A | Fracture of unspecified part of neck of right femur, initial encounter |
| Orthopedics | Trauma & Sports Injuries | M23.50 | Chronic instability of knee, unspecified knee |
| Orthopedics | Trauma & Sports Injuries | S62.101A | Fracture of unspecified carpal bone, right wrist, initial encounter |
| Orthopedics | Pediatric & Congenital | Q65.89 | Other specified congenital deformities of hip |
| Orthopedics | Pediatric & Congenital | Q74.0 | Other congenital malformations of upper limb(s) including shoulder girdle |
| Pulmonology | Airway & Obstructive Disease | J45.909 | Unspecified asthma, uncomplicated |
| Pulmonology | Airway & Obstructive Disease | J44.9 | Chronic obstructive pulmonary disease unspecified |
| Pulmonology | Airway & Obstructive Disease | J47.9 | Bronchiectasis uncomplicated |
| Pulmonology | Interstitial & Rare Lung Disease | J84.10 | Pulmonary fibrosis unspecified |
| Pulmonology | Interstitial & Rare Lung Disease | J67.9 | Hypersensitivity pneumonitis due to unspecified organic dust |
| Pulmonology | Interstitial & Rare Lung Disease | E84.0 | Cystic fibrosis with pulmonary manifestations |
| Pulmonology | Pulmonary Vascular & Critical Care | I27.20 | Pulmonary hypertension unspecified |
| Pulmonology | Pulmonary Vascular & Critical Care | I26.99 | Other pulmonary embolism without acute cor pulmonale |
| Pulmonology | Pulmonary Vascular & Critical Care | J96.90 | Respiratory failure unspecified unspecified whether with hypoxia or hypercapnia |

Supplementary Table 4 – Unique Vocabularies in each tier

| **Model** | **Tier** | **VocabSize** | **Diagnosis Codes** | **Procedure Codes** | **LAB Codes** | **Demographic Codes** |
| --- | --- | --- | --- | --- | --- | --- |
| Primarycare | Primary Care | 21 | 0 | 0 | 0 | 21 |
| Cardiology | Specialty | 46 | 0 | 0 | 0 | 46 |
| Dermotology | Specialty | 42 | 0 | 0 | 0 | 42 |
| ENT | Specialty | 46 | 0 | 0 | 0 | 46 |
| Endocrinology | Specialty | 43 | 0 | 0 | 0 | 43 |
| Gastroenterology | Specialty | 44 | 0 | 0 | 0 | 44 |
| Nephrology | Specialty | 42 | 0 | 0 | 0 | 42 |
| Neurology | Specialty | 44 | 0 | 0 | 0 | 44 |
| Oncology | Specialty | 46 | 0 | 0 | 0 | 46 |
| Orthopedics | Specialty | 44 | 0 | 0 | 0 | 44 |
| Pulmonology | Specialty | 44 | 0 | 0 | 0 | 44 |
| Cardiology_Arrhythmias_and_Electrophysiology | Subspecialty | 2123 | 1298 | 350 | 469 | 6 |
| Cardiology_Heart_Failure_and_Structural_Disease | Subspecialty | 1986 | 1290 | 279 | 412 | 5 |
| Cardiology_Ischemic_and_Vascular_Disease | Subspecialty | 2057 | 1297 | 283 | 472 | 5 |
| Dermotology_Hair_and_Nail | Subspecialty | 1844 | 1166 | 260 | 411 | 7 |
| Dermotology_Inflammatory_and_Autoimmune_Skin | Subspecialty | 1986 | 1363 | 234 | 383 | 6 |
| Dermotology_Skin_Cancer_and_Precancer | Subspecialty | 2323 | 1600 | 269 | 450 | 4 |
| ENT_Ear_and_Balance | Subspecialty | 2168 | 1557 | 214 | 389 | 8 |
| ENT_Nose_and_Sinus | Subspecialty | 2074 | 1390 | 239 | 438 | 7 |
| ENT_Throat_and_Head-Neck | Subspecialty | 1891 | 1235 | 265 | 387 | 4 |
| Endocrinology_Adrenal_and_Bone_Disorders | Subspecialty | 1930 | 1380 | 190 | 354 | 6 |
| Endocrinology_Diabetes_and_Metabolic | Subspecialty | 1987 | 1264 | 238 | 479 | 6 |
| Endocrinology_Thyroid_and_Pituitary | Subspecialty | 2158 | 1412 | 272 | 465 | 9 |
| Gastroenterology_Inflammatory_and_Autoimmune_GI | Subspecialty | 2059 | 1380 | 252 | 421 | 6 |
| Gastroenterology_Liver_and_Hepatology | Subspecialty | 2295 | 1416 | 328 | 543 | 8 |
| Gastroenterology_Pancreatic_and_Functional_GI | Subspecialty | 1891 | 1157 | 267 | 460 | 7 |
| Nephrology_Acute_and_Glomerular_Disorders | Subspecialty | 2024 | 1182 | 320 | 515 | 7 |
| Nephrology_Chronic_Kidney_Disease_and_Hypertension | Subspecialty | 1989 | 1181 | 300 | 501 | 7 |
| Nephrology_Stones_and_Transplantation | Subspecialty | 2050 | 1299 | 297 | 445 | 9 |
| Neurology_Epilepsy_and_Headache | Subspecialty | 2348 | 1457 | 358 | 528 | 5 |
| Neurology_Neurodegenerative_and_Demyelinating | Subspecialty | 2382 | 1558 | 306 | 512 | 6 |
| Neurology_Stroke_and_Vascular_Neurology | Subspecialty | 2167 | 1287 | 321 | 553 | 6 |
| Oncology_Hematology | Subspecialty | 1704 | 964 | 317 | 416 | 7 |
| Oncology_Oncology | Subspecialty | 1974 | 1308 | 287 | 371 | 8 |
| Oncology_Solid_Tumors | Subspecialty | 2007 | 1349 | 273 | 376 | 9 |
| Orthopedics_Joint_Disorders | Subspecialty | 2428 | 1673 | 329 | 420 | 6 |
| Orthopedics_Pediatric_and_Congenital | Subspecialty | 2496 | 1815 | 280 | 389 | 12 |
| Orthopedics_Trauma_and_Sports_Injuries | Subspecialty | 2079 | 1416 | 263 | 393 | 7 |
| Pulmonology_Airway_and_Obstructive_Disease | Subspecialty | 2447 | 1549 | 355 | 535 | 8 |
| Pulmonology_Interstitial_and_Rare_Lung_Disease | Subspecialty | 1978 | 1276 | 310 | 386 | 6 |
| Pulmonology_Pulmonary_Vascular_and_Critical_Care | Subspecialty | 2169 | 1409 | 302 | 452 | 6 |

Supplementary Table 5 - Tokenization and Input Construction

| **Parameter** | **Value** |
| --- | --- |
| Windowing strategy | Sliding window |
| Window size | 64 tokens |
| Window overlap | 4 tokens |
| Target mode | Next-token prediction (last token in window) |
| Special tokens | <s> (SOS), </s> (EOS), <pad> (PAD), <unk> (UNK) |

Supplementary Table 6 – Embedding Configuration

| **Parameter** | **Value** |
| --- | --- |
| Token embedding dimension | 512 |
| Type embedding categories | 5 (Demographic, Diagnosis, Procedure, Lab, Other) |
| Time embedding buckets | 1000 |
| ICD ontology augmentation | Hierarchical ancestor averaging with learnable scaling factor |

Supplementary Table 7 – Architecture Hyperparameters

| **Parameter** | **Value** |
| --- | --- |
| Model type | Decoder-only Transformer |
| Hidden dimension (d_model) | 512 |
| Number of layers | 4 |
| Attention heads | 8 |
| Feed-forward dimension | 2048 (4 × d_model) |
| Activation function | SwiGLU |
| Dropout rate | 0.2 |
| Stochastic depth (max rate) | 0.1 |
| Weight initialization | Xavier Uniform |

Supplementary Table 8 - Training Setup

| **Parameter** | **Value** |
| --- | --- |
| Optimizer | AdamW |
| Learning rate | 1 × 10⁻⁴ |
| Weight decay | 0.1 |
| Gradient clipping (max norm) | 1.0 |
| Label smoothing | 0.1 |
| Batch size | 128 |
| Maximum epochs | 500 |
| Early stopping patience | 10 epochs |
| Loss function | Cross-Entropy (non-padding tokens only) |
